# Transcriptional activation of zebrafish *fads2* promoter and its transient transgene expression in yolk syncytial layer of zebrafish embryos

**DOI:** 10.1038/s41598-018-22157-4

**Published:** 2018-03-01

**Authors:** Shu-Shen Tay, Meng-Kiat Kuah, Alexander Chong Shu-Chien

**Affiliations:** 1 School of Biological Sciences, Universiti Sains Malaysia, 11800 Minden, Penang Malaysia; 2 Centre for Chemical Biology, Universiti Sains Malaysia, Sains@USM, Block B No. 10, Persiaran Bukit Jambul, 11900 Bayan Lepas, Penang Malaysia

## Abstract

The front-end desaturases (Fads) are rate-limiting enzymes responsible for production of long-chain polyunsaturated fatty acids (LC-PUFA). The full spectrum of the transcriptional regulation of *fads* is still incomplete, as cloning of *fads* promoter is limited to a few species. Here, we described the cloning and characterisation of the zebrafish *fads2* promoter. Using 5′-deletion and mutation analysis on this promoter, we identified a specific region containing the sterol regulatory element (SRE) which is responsible for the activation of the *fads2* promoter. In tandem, two conserved CCAAT boxes were also present adjacent to the SRE and mutation of either of these binding sites attenuates the transcriptional activation of the *fads2* promoter. An *in vivo* analysis employing GFP reporter gene in transiently transfected zebrafish embryos showed that this 1754 bp upstream region of the *fads2* gene specifically directs GFP expression in the yolk syncytial layer (YSL) region. This indicates a role for LC-PUFA in the transport of yolk lipids through this tissue layer. In conclusion, besides identifying novel core elements for transcriptional activation in zebrafish *fads2* promoter, we also reveal a potential role for *fads2* or LC-PUFA in YSL during development.

## Introduction

In eukaryotic cells, the front-end desaturases (Fads) catalyse the introduction of a double bond at fixed number of carbons from the carboxyl group. A typical Fads possesses characteristic features such as three histidine-rich boxes, transmembrane regions, and an N-terminal cytochrome b_5_ domain containing the heme-binding motif HPGG. Long-chain polyunsaturated fatty acids (LC-PUFA) such as arachidonic acid (ARA, 20:4n-6), eicosapentaenoic acid (EPA, 20:5n-3) and docosahexaenoic acid (DHA, 22:6n-3) are generated from shorter polyunsaturated fatty acids (PUFA) through the actions of Fads and elongases (Elovl). LC-PUFA are crucial for maintenance of cellular membrane integrity, as precursors for eicosanoids, regulation of gene expression and signal transduction pathways. In aquaculture, the interest to decipher the activities of the LC-PUFA biosynthesis enzymes and transcription factors involve in LC-PUFA biosynthesis is driven by the pressing need to improve performance of vegetable oils in aquafeeds^1^. These oils are lacking in LC-PUFA but rich in C_18_ PUFA. Understandably, a greater understanding of distinctive bioconversion capacities of C_18_ PUFA to LC-PUFA in different farmed species could theoretically improve the strategy of employing vegetable oil as feeds^2^.

Among the vertebrates, numerous studies have reported the molecular cloning and characterisation of Fads from different fish species^1^. Fads from mammals are principally mono-functional, with Δ5 and Δ6 desaturations being carried out by two separate genes, *Fads1* and *Fads2*, respectively^3^. In fish, *fads1* have so far been isolated from only one species, a basal gnathostome, leading to the hypothesis that a complete loss of *fads1* in teleosts have occurred following gnathostome radiation^4^. Isolation and characterisation of teleost *fads2* to date have reported a broad range of substrate specificities, with Δ4, Δ5, Δ6 and Δ8 desaturation capacities reported. In addition, both mono-functional and bi-functional *fads2* have been isolated from a myriad of teleost species^5–7^. As compared to freshwater species, marine teleosts seemed to display a lesser capacity for bioconversion of C_18_ PUFA to LC-PUFA, due to the loss of crucial *fads* from its genome or low activities of *fads2* or *elovl*^8^. This was postulated to be a result of richer LC-PUFA content in the marine environment, causing diminished capacity for LC-PUFA biosynthesis. Subsequent works however advocated the influence of feeding niche on LC-PUFA biosynthesis, when a catalogue of *fads2* and *elovl* were isolated from marine fish species which possess a diet with limited DHA intake^6,9^.

Despite the multiple interests in molecular characterisation of *fads* in teleost, there is a paucity of understanding on its regulation at the transcriptional level. It is conceivable that differences in regulatory activities by dissimilar transcription factors responding to different cues including nutritional status is responsible for the different LC-PUFA biosynthesis capacity in various fish species. These transcription factors themselves are subjected to regulation by the nutritional status of the animal. Therefore, efficient use of vegetable oils in aquafeeds will benefit from the ability to decipher the link between the overall lipid metabolic pathways modification caused by the diet and the subsequent influence on lipid homeostasis genes at the transcriptional level. To date, promoter sequences from teleost *fads2* have been isolated from Atlantic salmon^10^, Atlantic cod^10^, rainbow trout, European sea bass^11^, Japanese seabass^12^, large yellow croaker^13^ and rabbit fish^14^. Firstly, these works collectively showed the presence of binding sites for transcription factors known to play a role in mammalian cholesterol and lipogenic synthesis pathways such as sterol regulatory element-binding protein (Srebp), nuclear factor Y (NF-Y) and ubiquitous transcription factor Sp1 in the *fads2* promoter^10–14^. Secondly, these studies suggested an interplay between different transcription factors to mediate the regulation of *fads2* transcription.

The zebrafish has gained reputation as a useful model in understanding the role of lipids and fatty acids during development^15,16^. Largely driven by the existence of multiple homologous genes involved in conserved lipid and lipoprotein metabolism network, zebrafish have been used to gain insight on lipid adsorption, atherosclerosis, fatty liver disease and obesity^15,17^. Zebrafish tissues are reported to contain significantly higher concentrations of PUFA than equivalent mammalian tissues^18,19^. Molecular cloning, functional characterisation and expression profile of *fads2* and several *elovl* genes during embryogenesis have been reported in zebrafish^5,20–23^. However, our knowledge on the function of *fads2*/*elovl* and LC-PUFA during development is still fragmentary. Given how important transcriptional regulation in influencing the activities of *fads2* may be and how little is known about the regulators and cellular conditions for expression, it is opportune to use zebrafish as a model to fill these gaps.

In this present study, we described the cloning and characterisation of the zebrafish *fads2* promoter. This includes identification and functional characterisation of pivotal regulatory elements responsible for driving transcriptional activities using luciferase reporter gene in a zebrafish cell line. In addition, we also carried out an *in vivo* characterisation of the zebrafish *fads2* promoter using a transient approach to track its expression in developing zebrafish embryos.

## Results

### Cloning and characterisation of the zebrafish *fads2* promoter

We isolated a putative promoter region of 1754 bp, comprising 19 bp of the 5′-UTR of zebrafish Δ5/ Δ6 *fads2*^5^ and 1735 bp of the upstream region. For positioning purpose, +1 was given to the putative transcription start site (TSS) derived from comparison of the 5′-end of full-length cDNA (GenBank accession number: NM_131645.2) and DNA sequences of the *fads2* gene. Within this *fads2* promoter region, a classic transcription initiation element, the TATA box was identified at the location of −26 bp (Supplementary data 1).

### Zebrafish Srebp upregulates *fads2* promoter activity

**Figure 1:**
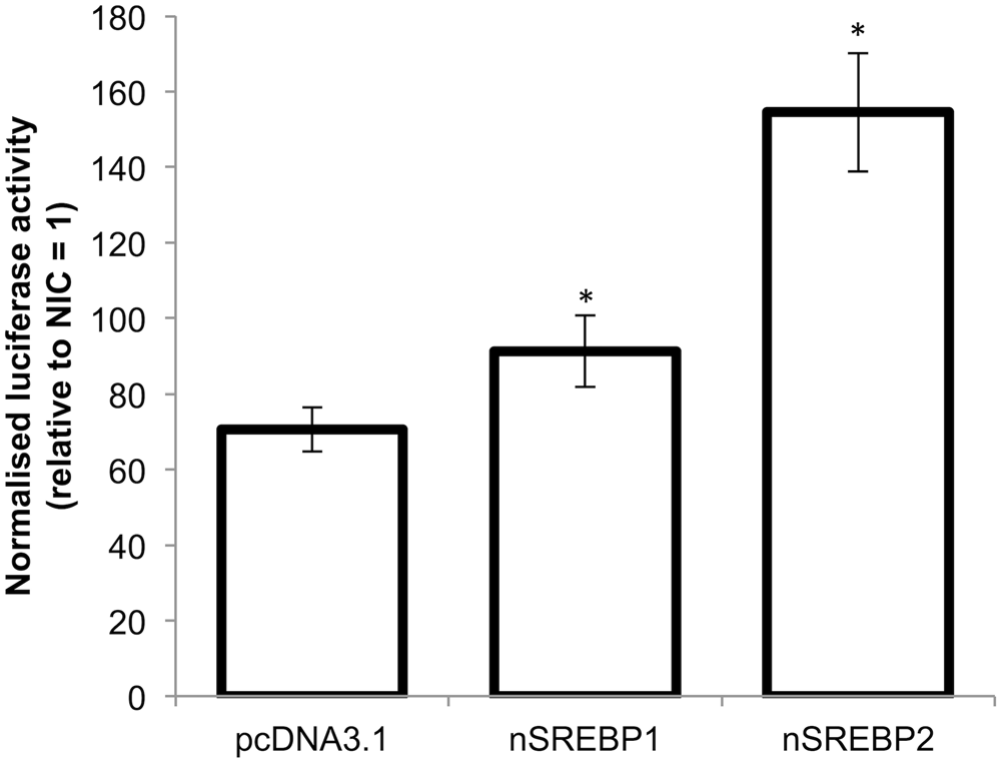
Effects of nSrebp proteins on the zebrafish *fads2* promoter activation. ZFL cells were co-transfected with nSrebp1 or nSrebp2 expression plasmid (empty pcDNA3.1 expression plasmid as control), and pGL3 promoter-luciferase reporter plasmid fused with the zebrafish *fads2* desaturase promoter (−1735/+19 bp). Results represent normalised luciferase activity (to *Renilla* luciferase activity) of *fads2* promoter in the ZFL cells relative to empty pGL3-Basic plasmid. Values are means ± SD (n = 3); *P* < 0.05 by one-way ANOVA.

Co-transfection of ZFL cells with the *fads2*-1735 promoter-luciferase reporter plasmid and expression plasmid containing either the nuclear form of zebrafish Srebp1 (nSrebp1) or Srebp2 (nSrebp2) proteins, respectively, resulted in significantly higher reporter activities than cells transfected with empty pcDNA3.1 expression plasmid (Fig. 1). Among the two isoforms, the zebrafish nSrebp2 protein resulted in higher luciferase reading. This signified the importance of Srebp proteins in driving the expression of zebrafish *fads2*, and subsequent luciferase reporter transactivation assays were performed with the overexpression of zebrafish nSrebp2 protein.

### Identification of *cis*-regulatory elements within the −214/−67 bp region of zebrafish *fads2* promoter

**Figure 2:**
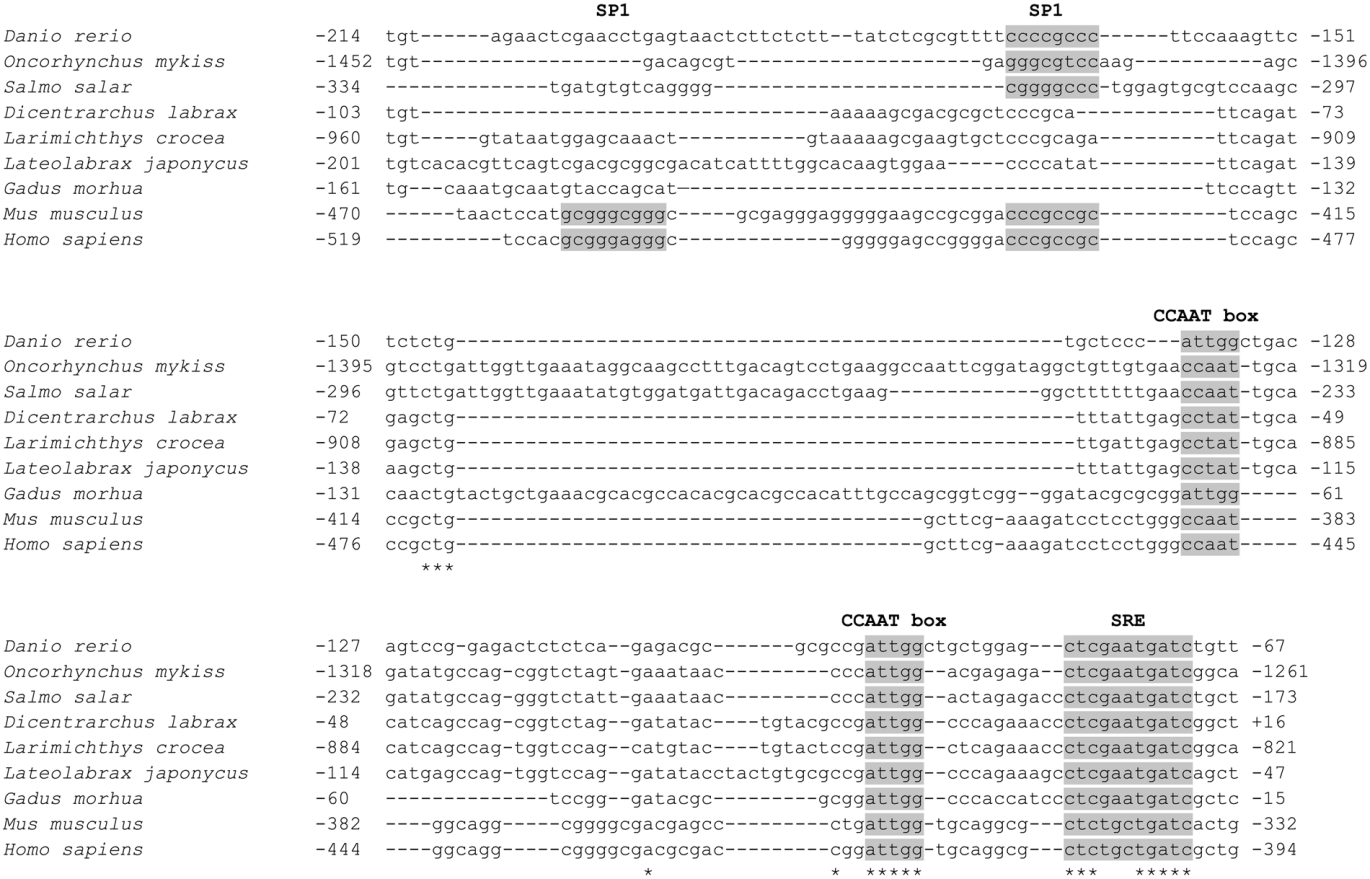
Conserved *cis*-regulatory elements within the core promoter regions of the *fads2* genes from zebrafish and other vertebrate counterparts. Sequence alignment was performed with web-based program, MAFFT (version 7). Numbers indicating the sequence positions are relative to TSS (+1), based on the cDNA sequence information deposited in the GenBank (*Danio rerio*: CU694371.13; *Oncorhynchus mykiss*: KT781408.1; *Salmo salar*: AY736067.2; *Dicentrarchus labrax*: FP671139.1; *Larimichthys crocea*: KT781409.1; *Lateolabrax japonycus*: KT781410.1; *Gadus morhua*: FJ859898.1; *Mus musculus*: AC135670.3 and *Homo sapiens*: AP002380.3). Conserved regions for the SP1 binding site, CCAAT boxes and SRE consensus sequence are highlighted in grey, while identical nucleotides are indicated with asterisks.

To identify the presence of any potential *cis*-regulatory element, the nucleotide sequence within the −214/−67 bp region of zebrafish *fads2* promoter was aligned and compared to corresponding sequences from rainbow trout^13^ (GenBank accession number: KT781408.1), Atlantic salmon^10^ (GenBank accession number: AY736067.2), European seabass^11^ (GenBank accession number: FP671139.1), large yellow croaker^13^ (GenBank accession number: KT781409.1), Japanese seabass^12^ (GenBank accession number: KT781410.1), Atlantic cod^10^ (GenBank accession number: FJ859898.1), human^24^ (GenBank accession number: AP002380.3) and mouse (GenBank accession number: AC135670.3). The alignment disclosed the presence of several conserved putative transcription factor binding sites, namely two inverted CCAAT boxes, an inverted ubiquitous transcription factor SP1 site and a sterol regulatory element (SRE) within the cloned promoter region (Fig. 2). The result also showed that the CCAAT boxes were highly conserved across the mammalian and fish species while the SRE (CTCGAATGATC) found in the zebrafish *fads2* promoter was identical to those in all the teleost species but differed from the mammalian SRE (CTCTGCTGATC) by three consecutive nucleotides. The SP1 binding site is less conserved amongst the mammalian and fish species, with two SP1 binding site in the mammalian *Fads2* promoters, while teleost *fads2* promoters typically contain either a single SP1 site or none.

### −161/−67 bp region is critical for zebrafish *fads2* promoter activation

**Figure 3:**
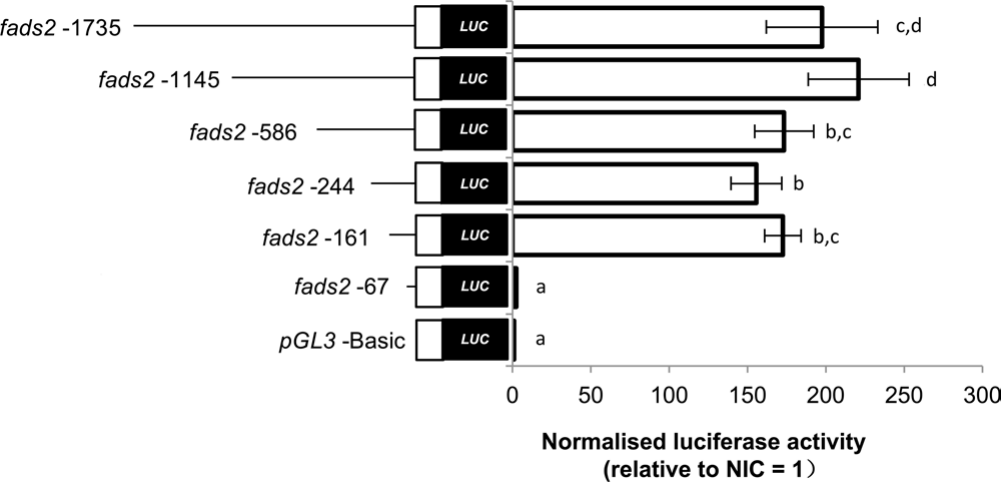
5′-deletion analysis of the zebrafish *fads2* promoter. ZFL cells were transiently co-transfected with the *fads2* 5′-deletion promoter-luciferase reporter plasmids, nSrebp2 expression plasmid together with *Renilla* luciferase reference plasmid pRL*-*SV40. 5′-deletion plasmids were named according to respective position in relation to TSS (+1) and are represented by horizontal line on the left. Non-coding exon is indicated with open boxes. Luciferase activity of *fads2* promoter in ZFL cells is expressed as normalised luciferase activity (to *Renilla* luciferase activity) relative to empty pGL3-Basic plasmid. Values are means ± S.D. (n = 3) (right panel). Groups indicated with different letters are significantly different (Tukey’s test; *P* < 0.05).

We sought to determine a minimal *fads2* promoter fragment capable of activating transcription by fusing a series of 5′–deleted promoter fragments to the luciferase reporter gene. Upon normalisation to values obtained with a promoter-less pGL3.1 plasmid, the intact *fads2* promoter (−1735/+19 bp) drove a luciferase activity of approximately 200-fold (Fig. 3). Deletion of promoter up to 161 bp upstream of the TSS did not greatly diminish transcription activities, notwithstanding some reduction in luciferase readings as compared to the longer fragments. However, further deletion of the sequence within −161/−67 bp almost completely abolished the promoter activity, which indicated a mandatory site within the region of −161 to −67 bp for activation of the *fads2* activities. This region contains both CCAAT boxes and the SRE (Supplementary data 2). This also means the removal of the SP1 was not detrimental to the activation of zebrafish *fads2* promoter.

### CCAAT boxes and SRE mediates *fads2* promoter activity

**Figure 4:**
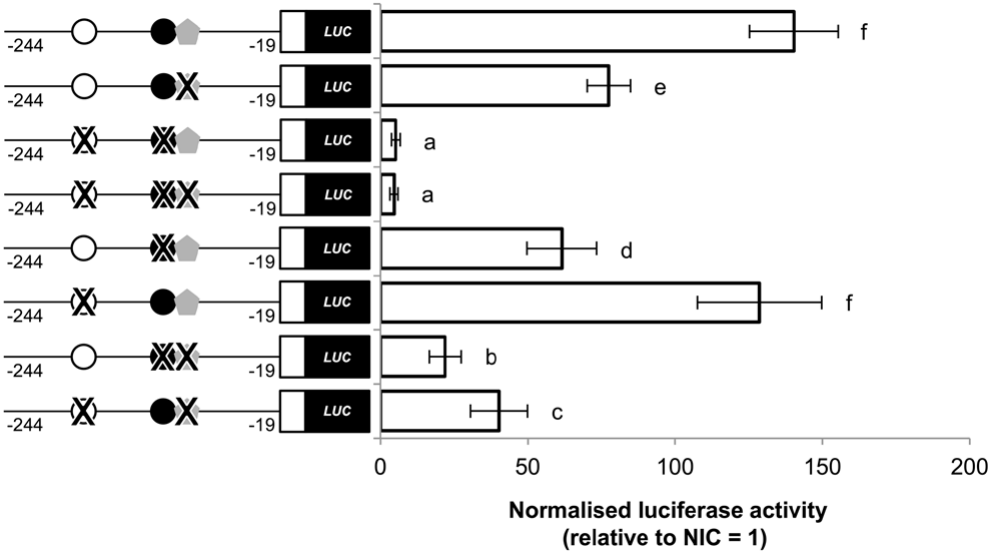
Mutated CCAAT box and SRE reduce transcriptional activity of the zebrafish *fads2* promoter. Single or multiple site mutations (**X**) of SRE (), CATp (●) and CATd (○) were introduced into the *fads2*−244 plasmid. ZFL cells were transiently co-transfected with various mutated *fads2*−244 plasmids, nSrebp2 expression plasmid, together with *Renilla* luciferase reference plasmid pRL-SV40. Luciferase coding region is indicated by shaded box and activity of the *fads2* promoter in ZFL cells is expressed as normalised luciferase activity (to *Renilla* luciferase activity) relative to the empty pGL3-Basic plasmid. Values are means ± SD of three independent experiments (n = 3) (right panel). Groups indicated with different letters are significantly different (Tukey’s test; *P* < 0.05).

We introduced single or multiple site mutations for the SRE, proximal CCAAT box (CATp) and distal CCAAT box (CATd) to the *fads2*-244 promoter-luciferase reporter plasmid, followed by the dual luciferase reporter assay in ZFL (Fig. 4). Firstly, results showed that both CATp and CATd could drive expression of promoter, as seen in the fragment with mutated SRE, although the expression was lower than the fragment with all three binding sites intact. In comparison, disrupting both proximal and distal CCAAT bindings sites resulted in total abolishment of luciferase expression, an observation which was recapitulated when all three binding sites were mutated. This implies an obligation for both CCAAT and SRE to optimally drive zebrafish *fads2*, although the former was still able to activate a reduced measure of transcriptional activities. When SRE was present, CATp seemed to play a more prominent role as compared to CATd in driving promoter activation. A similar pattern was also observed when SRE was disrupted.

### *In vitro* binding of ZFL nuclear proteins at the SRE and CCAAT regions in the zebrafish *fads2* promoter

**Figure 5:**
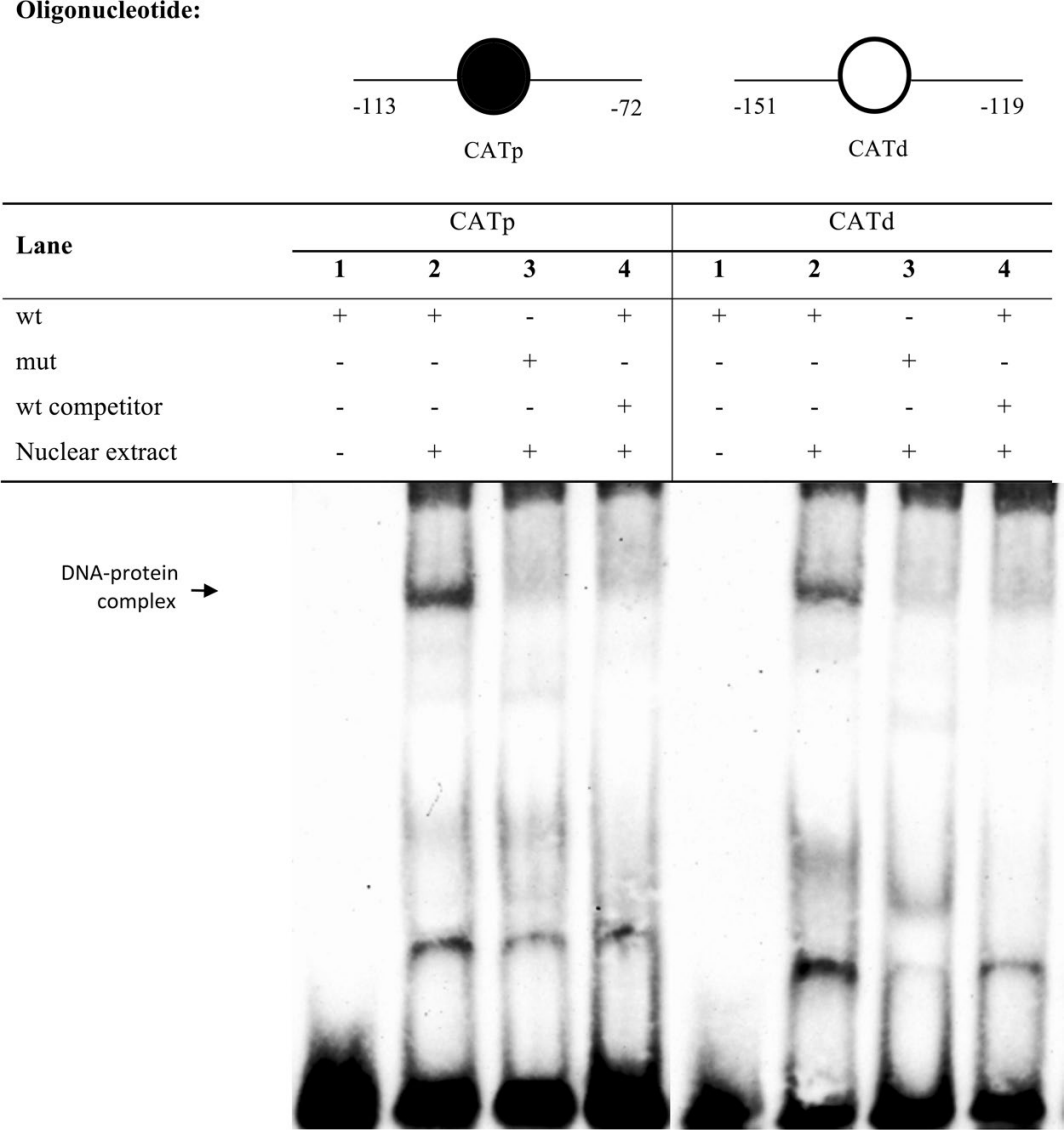
Binding of ZFL nuclear proteins to the proximal CCAAT box (CATp) and distal CCAAT box (CATd) on the zebrafish *fads2* promoter. EMSA was performed with biotin-labelled oligonucleotides containing CATp or CATd box and nuclear extract of ZFL cells. Specific binding of DNA-protein complex was validated with site-mutated oligonucleotides (Lane 3) and 100-fold molar excess of non-labelled competition oligonucleotides (Lane 4).

Based on the observations from the mutagenesis experiments, we ran EMSA to assess the binding capacities of transcription factors in ZFL nuclear extract to the *fads2* promoter *cis*-regulatory elements. Results showed the formation of DNA-protein complex when ZFL cells nuclear extract was incubated with wild type oligonucleotide probe containing CATp binding site. There was no complex formed when the probe was incubated without the nuclear extract or when a probe with mutated CATp sequence was used with the nuclear extract. Similarly, the use of non-labelled wild type oligonucleotides in excess amount completely outnumbered the formation of labelled DNA-protein complex. A comparable pattern was shown with CATd sequence (Fig. 5).

**Figure 6:**
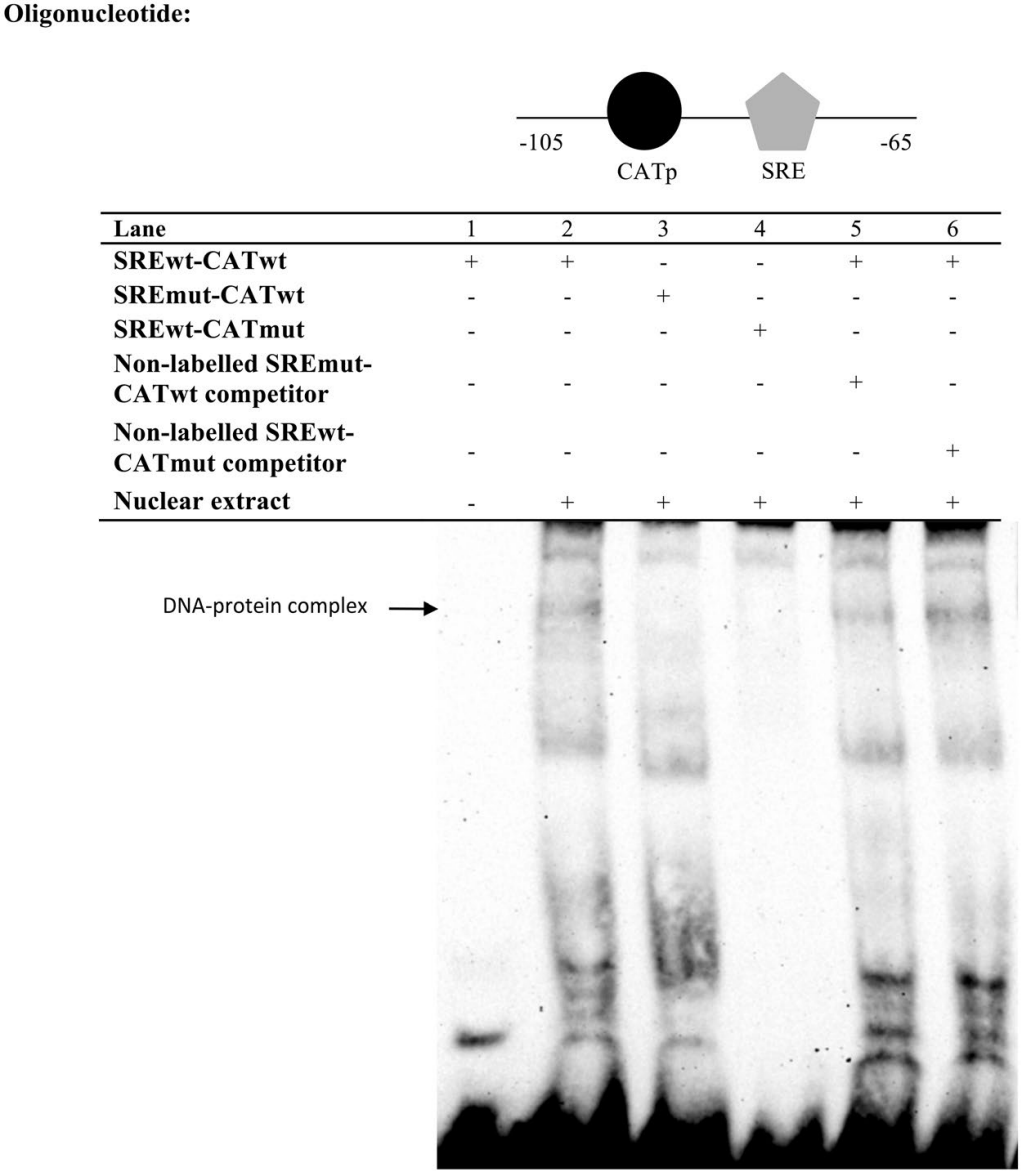
Binding of ZFL nuclear proteins to the SRE on the zebrafish *fads2* promoter. Biotin-labelled probes containing SRE and proximal CCAAT box (CATp) were incubated with the nuclear extract of ZFL cells. Specific binding of DNA-protein complex was validated with site-mutated oligonucleotides (Lanes 3 and 4) and 100-fold molar excess of non-labelled mutated competition oligonucleotides (Lane 5 and 6).

Unlike the CATp and CATd probes, DNA-protein complex was not observed when nuclear proteins were added into the sample containing oligonucleotides with only SRE site (result not shown). Taking this and the results from mutagenesis experiment into consideration, we used a longer oligonucleotide consisting of both SRE and the adjacent CATp. Results showed that binding of transcription factors and DNA occurred with the simultaneous presence of both the SRE and CATp binding sites (Fig. 6). Mutation on either site prevented the formation of any DNA-protein complex. When the wild type labelled probe was incubated with excessive non-labelled probe containing mutated SRE and intact CATp, a decrease in DNA-complex staining was observed due to competition for binding. This result encored the observation in cell-based luciferase assay which showed the requirement of CATp for activation of reporter gene expression. In contrast, when wild type labelled probe was incubated with excessive non-labelled probe with intact SRE and mutated CATp binding site, the formation of DNA-complex occured. Therefore, in the absence of any conjugated protein binding to CATp, the binding of Srebp to the SRE was perturbed. Taken together, the results from mutagenesis and EMSA suggested a functional role for CCAAT box in the Srebp dependent transcriptional activation of zebrafish *fads2* promoter.

### Transient expression of *fads2* promoter (−1735/+19 bp) in developing zebrafish embryos

**Figure 7:**
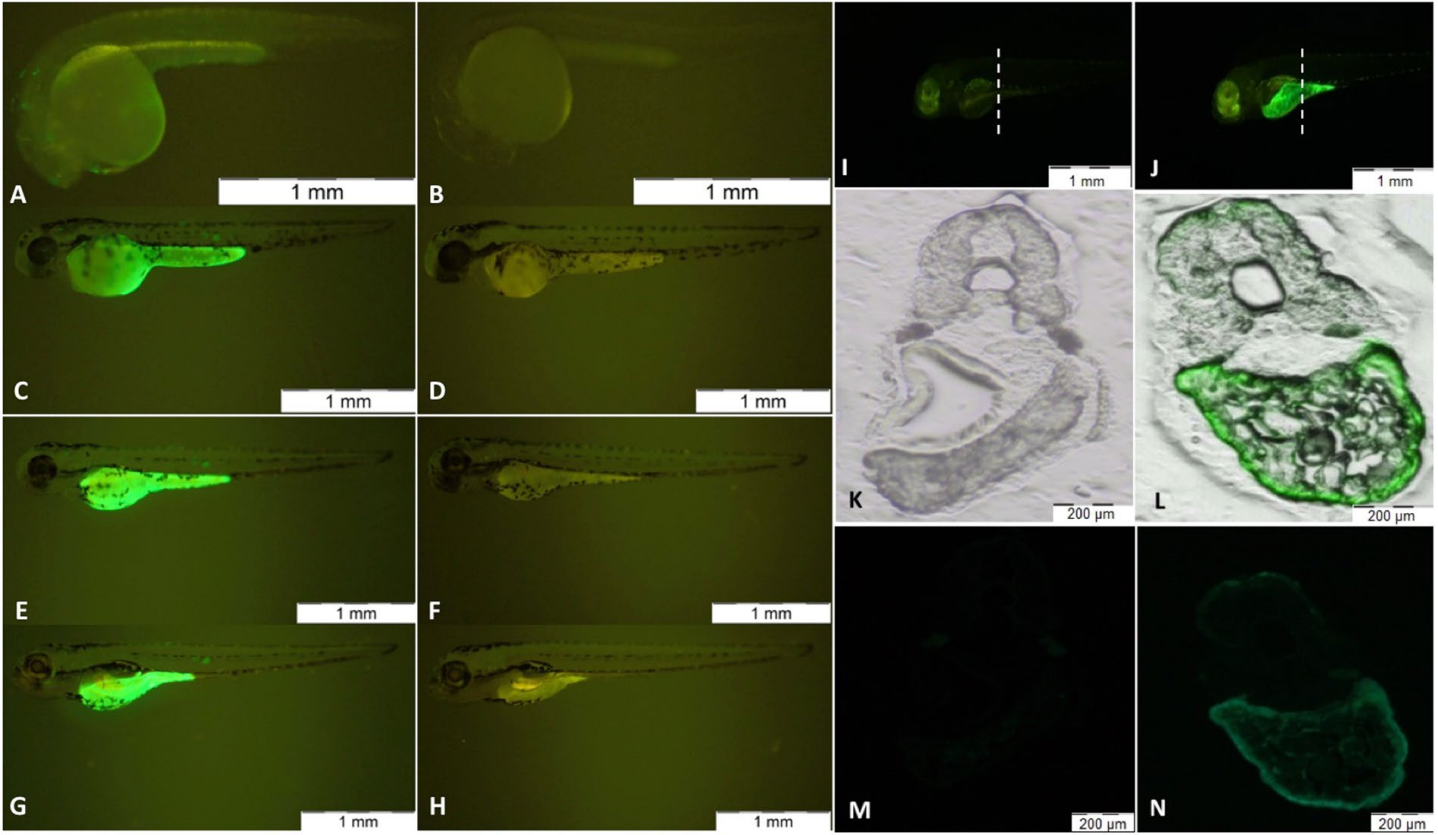
Transient GFP expression of linearised *fads2* promoter (−1735/+19 bp) localised in yolk syncytial later (YSL) of zebrafish embryos. Lateral view of 26, 48, 72 and 96 hpf of transient transgene embryo injected with linearised *fads2* promoter (−1735/+19 bp) showing increased expression in YSL (**A**,**C**,**E**,**G**) and their corresponding control (**B**,**D**,**F**,**H**). Cross-sectional view of a 120hpf control embryo (**K**–**N**) and a 120hpf transient transgenic embryo (**I**,**J**) injected with linearised *fads2* promoter (−1735/+19 bp) showing expression at YSL.

We observed GFP signal in the yolk syncytial layer (YSL) of embryos beginning at 26 hpf for both the pZs−244 bp and pZs−1735 bp constructs, respectively. As embryos developed, higher percentage of embryos with YSL-GFP expression were observed, along with increase in GFP signal intensity (Fig. 7). At 120 hpf, more than 95% of injected embryos showed GFP expression at the YSL (Table 1). Therefore, a *fads2* promoter fragment containing response elements such as SRE, CATp and CATd proven to be essential for activation of expression is now shown to drive a spatio-temporal expression of *fads2* in the zebrafish YSL.

**Table 1:** Transient expression of the linearised *fads2* promoter (−1735/+19 bp) in zebrafish embryos. Figures are cumulative embryos scored from three independent microinjections at 120 hpf.

Description	Embryo number (%)
Injected	72
Survival	63 (87.5%)
Dead/Abnormal	9 (12.5%)
GFP expressing embryos	58 (92.1%)
**Localisation of GFP**	
YSL	57 (98.3%)
Non-specific (notochord)	1 (1.7%)

## Discussion

The zebrafish *fads2* was first cloned and shown to have the capacity for *in vitro* Δ5/Δ6 desaturation^5^. The transcripts were reported to express in several tissues known for biosynthesis of LC-PUFA during embryonic development^21^. The mRNA transcript of zebrafish *fads2* is also seemingly regulated by dietary PUFA intake^25^. Despite these studies, investigation on the regulation of *fads2* expression at transcription level is lacking. Here, we cloned a 1754 bp promoter region of the zebrafish *fads2* gene to determine response elements critical for control of *fads2* expression. We showed here that like all known teleost *fads2* promoters, zebrafish *fads2* promoter harbours SRE and CCAAT binding elements^10–13^. Zheng *et al*.^10^ also showed a high degree of sequence conservation between mammalian, amphibian and teleost species, which indicates conserved mechanism of *fads2* role. As with salmon^10^ and trout^13^, putative SP1 binding site was also identified in this region of zebrafish *fads2* promoter. In contrast, all sequenced promoters of *fads2* from marine teleost species so far do not contain SP1 element, which was postulated as a reason for lower *fads2* expression in marine fish species^11–13^.

Results from the promoter deletion experiment demarcated regions responsible for transcriptional activation of the zebrafish *fads2*. One such region is the *cis-*element SRE, the binding site for Srebp transcription factors. Using mutagenesis approach, we further corroborate the obligation of the SRE region in activating transcription of zebrafish *fads2*. We also showed that this sequence forms a DNA-protein complex with nuclear extract of ZFL cells. In human, an E-box like SRE region, as opposed to the classical SRE region, was crucial for both activation and suppression of the FADS2 gene^24^. Srebps are prominent regulators of lipid, fatty acid and cholesterol biosynthesis, and were first identified as nuclear proteins possessing binding abilities to SRE of low density lipoprotein receptor^26^. Like mammals, fish have two distinct homologues of mammalian Srebp1 and Srebp2^27^. In zebrafish, activation of hepatic Srebp induce the expression of both lipid and cholesterol biosynthesis genes^28^. Our results also showed that in ZFL cells, Srebp2 activated a higher level of *fads2* transcription as compared to Srebp1. This is reminiscent of the findings in Atlantic salmon, where Srebp2 promoted higher *fads2* activities as compare to Srebp1^29^. The importance of Srebp in mediating teleost *fads2* expression has been shown through the use of cell-based luciferase assays involving Srebp expression plasmids or mutation of the promoter’s SRE response element^13,14,29^. In terms of tissue localisation, there was a parallel pattern between the distribution of *srebp* and *fads2* mRNA expression^27,30^. The role of Srebp as a sensory mediator between dietary LC-PUFA intake and LC-PUFA biosynthesis was also observed in several fish species wherein limited dietary LC-PUFA intake led to increased Srebp level, subsequently resulting in higher *fads2* mRNA expression^13,29–32^. In rat and human, dietary PUFA inhibits the rate of *Fads2* transcription through Srebp, probably by lessening the production of mature Srebp or accelerating the decay of *Srebp* mRNA^24,33^. Besides *FADS2*, Srebp have also been shown to regulate *ELOVL*^24,34^.

Through a combination of deletion and mutagenesis, we demonstrate the importance of two CCAAT motifs in the core promoter region of zebrafish *fads2* for transcriptional activation. The CCAAT box, named after the five nucleotides customarily found in the binding sites, is one of the most ubiquitous binding element in eukaryotic promoters, with several corresponding binding proteins already isolated and characterised^35^. The NF-Y is the major protein recognizing the CCAAT box, and is obligatory for the transcription of a considerable number of genes with ubiquitous or tissues-specific expression pattern^35^. Functional CCAAT motifs recognised by NF-Y are among the most frequent boxes found in promoters^36^. Mutation of putative NF-Y binding sites in *fads2* promoter of two marine teleost species also resulted in weaker transcriptional activities^10,14^.

Our present finding suggests that the putative NF-Y binding sites are required for formation of complex between Srebp and SRE, which then activates the transcription of reporter gene. This result resonated with earlier findings wherein Srebp was incapable of driving transcription of target genes if adjacent NF-Y binding sites were mutated^37–39^. By themselves, Srebp proteins are inherently weak transcriptional activators, which underlines the need for Srebp proteins to interact with additional transcriptional factors to regulate the expression of lipogenic genes^38,40,41^. Studies on promoters of cholesterol and fatty acid metabolism genes consistently highlight the role of NF-Y as a pivotal partner in Srebp-mediated regulation of transcriptional activities^36,38,41–43^. Elsewhere, studies on murine stearoyl-CoA desaturase (*Scd*) and human *FADS2* promoters both showed a requirement for putative NF-Y binding sites located adjacent to SRE for transcriptional activation^24,39^. Promoters of teleost and amphibian *fads* genes also contain a conserved region bearing NF-Y and Srebp binding sites at similar positions relative to the transcriptional start site (TSS)^10^. It is speculated that direct interaction of Srebp and NF-Y, together with the binding of both these transcription factors to neighbouring sites on the DNA lend stability to the DNA-protein complex^39^. In addition, our results showed that removal of the putative SP1 binding region did not supress the luciferase expression. The interaction between Srebp and SP1 in the regulation of *Fads* have not been demonstrated this far^24,33^, although a synergistic type of activation between these two transcriptional factors have been elucidated in other lipid metabolism genes^44^. A role for SP1 in Srebp-mediated activation of *Fads2* however cannot be ruled out as different isoforms of Srebp or nutritional conditions may utilise different co-regulatory factors to activate expression of *Fads*, as shown in promoters of fatty acid synthase^45^.

Fatty acids from yolk storage are critical for energy and structural components for both fish embryo and larval stages^46^. Together with the head region, yolk sac has been reported to contain majority of the lipids during zebrafish embryonic stages^19^. While saturated and monounsaturated fatty acids (SFA, MUFA) are consumed for energy, LC-PUFA are essential for cellular membrane integrity, regulation of gene transcription, regulation of signalling pathways and production of eicosanoids. Previously, the yolk was regarded as a non-metabolic active site for nutrient reserve, where molecules such as lipids are stored within yolk cells and transported when needed^46,47^. However, emerging findings employing zebrafish as the model organism, are recognising the yolk as site for active lipid metabolism preceding transportation^16^. Using a transient transgenic approach in zebrafish embryos, we showed that the −1735/+19 bp region of the *fads2* promoter directed a specific expression in the YSL beginning at 26 hpf embryos. In support of this, *fads2* and *elovl* transcripts were reported to be expressed in YSL at the 24 hpf stage, alongside brain tissues^18,21^. During early embryonic development, some of the blastomeres situated at surface of the yolk collapse and fuse with the yolk cells, leading to the formation of YSL^48,49^. It then undergoes a series of changes in terms of thickness and nuclei distribution, before going into the process of degradation upon the exhaustion of yolk supplies^50^. YSL governs major roles during development, including yolk metabolism, epiboly, specification of germ layers, organogenesis of endoderm organs and innate immunity^50–52^. A major role of YSL in teleost development is on the transport of nutrients from yolk to embryonic cells and tissues. Transcripts of genes related to lipid metabolism and transportation^53–58^, are expressed in YSL during early development to facilitate the hydrolysis and transport of yolk lipids. When these genes are disrupted, embryos show reduced yolk consumption, delayed embryonic growth and failure in lipid absorption^58,59^.

The presence of GFP signal in YSL also suggested a role for LC-PUFA, the product of *fads2* promoter activities, in transportation of yolk lipids. Detailed analyses of lipid classes in zebrafish yolk imply active *de novo* synthesis of lipids, in which fatty acids are resynthesised into major lipid classes between 0–48 hpf stage, followed by packaging into very low-density lipoproteins for supply to whole embryo beyond 48hpf^15,19^. In *Caenorhabditis elegans*, transportation of yolk lipoprotein to oocytes was disrupted in mutants with PUFA deficiency, a malaise restored by feeding worms with n-6 PUFA^60^. These findings, viewed alongside our results showing localisation of *fads2* promoter activity at the YSL, provides the collective interpretation that LC-PUFA is required for transportation of yolk lipids during development.

In adult teleost, the liver is a major site for LC-PUFA biosynthesis, with both *fads* and *elovl* transcripts being reported in adult zebrafish liver and early larval stages^18,21^. Since the injected embryos only showed expression in the YSL, the *fads2* promoter fragment lacks the regulatory region responsible for directing expression of *fads2* in other organs such as liver, intestine or brain. It is plausible that the regulatory elements for the expression of these tissues are located in more distal regions or/and introns^61,62^. Multiple studies have shown that recapitulation of endogenous expression by a promoter in zebrafish embryos depends on the length of the promoter fragment^62–64^.

In conclusion, we successfully isolated a 1754 bp upstream region of the zebrafish *fads2* promoter and identified several response elements critical to drive the activation of transcription. We also showed the capacity of this fragment to direct GFP expression at the YSL region of 26 hpf and older stages embryos. Since zebrafish relies on yolk as sole lipid source prior to onset of exogenous feeding capacity, it potentially provides a platform to further elucidate the role of *fads2* or LC-PUFA in transportation of yolk lipids across the YSL.

## Materials and Methods

### Cell line maintenance and subculture

The zebrafish liver cell line, ZFL (ATCC® CRL-2643™) was purchased from the American Type Culture Collection (ATCC, USA) and maintained in the complete growth medium at 28 °C following the protocol of ATCC. Routine subculture was performed once the ZFL cells have achieved 80 to 90% confluence in a 25 cm^2^ flask.

### Zebrafish maintenance and embryo collection

Wild type zebrafish (AB line) were purchased from the Institute of Molecular and Cell Biology (IMCB, Singapore), and maintained in the ZebTEC Stand Alone System (Tecniplast, USA). The fish were kept on a 13:11 h light/dark cycle at 28.5 °C, and fed till visual satiation twice daily with a combination of commercial micro pellet (Aquadene, Malaysia), frozen bloodworms and *Artemia* nauplii. Breeding and embryo collection were carried out according to Westerfield (2000) with adaptation^65^.

### Animal ethics approval

Husbandry, handling and use of animals in this manuscript comply with the guidelines and requirements of the Animal Ethics Committee, Universiti Sains Malaysia and were approved by the same committee (PA/ACSC/002/2011).

### Zebrafish *fads2* promoter cloning and promoter-luciferase reporter plasmid construction

A promoter region of 1754 bp, corresponding to −1735/+19 bp relative to the transcription start site of the zebrafish *fads2* gene was amplified with PCR utilising the *i*-Taq™ Plus DNA Polymerase (iNtRON, Korea). DNA extracted from the ZFL cells was used as the template for the amplification, together with the forward primer F1735 containing the restriction site for *Kpn*I, and a reverse primer R19 containing the restriction site for *Xho*I (Supplementary data 3). The reaction mixtures were subjected to a thermal cycling programme consisted of an initial denaturation step for 2 min at 94 °C, followed by 40 cycles of denaturation for 20 s at 94 °C, annealing for 20 s at 50 °C, and extension for 1 min/kb of product length at 72 °C, and a final extension step for 5 min at 72 °C. The resulted PCR products were gel purified, cloned into the pGL3-Basic luciferase reporter vector (Promega, USA) and sequenced. Similarly, for the 5′-deletion analysis, another five promoter-luciferase reporter plasmids containing the *fads2* promoter region with deletions of different lengths at the 5′-end were generated by using a series of forward primers (F1145, F586, F244, F161 or F67) and a common reverse primer (R19).

### Zebrafish Srebp expression plasmid construction

Total RNA was extracted from the adult zebrafish liver using the TRIzol^®^ Reagent (Invitrogen, Germany) according to the manufacturer’s descriptions. First-strand cDNA was generated via reverse transcription PCR using the M-MLV Reverse Transcriptase (Promega, USA). PCR fragments corresponding to the N-terminal of zebrafish *srebp-1* (nSrebp-1, 1-457 aa; NM_001105129.1) and *srebp-2* (nSrebp-2, 1-464 aa; NM_001089466.1) were identified through alignment with the human *SREBP* homologues (Nakakuki *et al*., 2007), and amplified from the first-strand cDNA using forward primers containing the restriction site for *KpnI* and reverse primers containing a stop codon (TAG) for translation termination and the restriction site for *XhoI* (Supplementary data 4). PCR amplification was performed as abovementioned and the resulted PCR products were cloned into the CMV promoter-driven expression plasmid pcDNA3.1 (Invitrogen, Germany).

### Transient transfection and dual luciferase reporter assay

Prior to the day of transient transfection, 2.5 × 10^4^ ZFL cells per well were seeded in a 96-well clear bottom white walled plates (Thermo Scientific, USA) and incubated overnight for cell attachment. Transient transfection was carried out using transfection reagent Lipofectamine™ 2000 (Life Technologies, Germany) at a volume of 0.15 µl per 100 ng DNA. For nSrebp overexpression assay, ZFL cells were co-transfected with *fads2*-1735 promoter-luciferase reporter plasmid (100 ng/well), one of the nSrebp expression plasmids (50 ng/well) and *Renilla* luciferase reference plasmid, pRL-SV40 (50 ng/well). ZFL cells prepared for the 5′-deletion assay were co-transfected with one of the pGL3 promoter-luciferase reporter plasmids (equimolar to 100 ng *fads2*-1735 plasmid/well), pcDNA3.1-nSrebp2 (50 ng/well) and pRL-SV40 (50 ng/well). Co-transfection for mutation analysis was performed with one of the mutated pGL3 promoter-luciferase reporter plasmids (100 ng/well), pcDNA3.1-nSrebp2 (50 ng/well) and pRL-SV40 (50 ng/well). Lastly, nuclear extracts for EMSA (refer to section 2.7) were prepared from the ZFL cells seeded in a 25 cm^2^ flasks and transfected with pcDNA3.1-nSrebp2 (2 µg/ml). Briefly, plasmid DNA-Lipofectamine™ 2000 complexes in 50 µl Opti-MEM were equally distributed to each well and the ZFL cells were incubated for 4.5 h at 28.5 °C. The plasmid DNA-Lipofectamine™ 2000 complexes contained-culture medium was then replaced with fresh culture medium and the transfected ZFL cells were further incubated for 24 h before being proceeded to subsequent assay. The ZFL cells were harvested in 20 µl/well Passive Lysis Buffer and luciferase assay was carried out using the Dual-Glo® Luciferase Assay System (Promega, USA) according to the manufacturer’s protocols. All the luminescence measurements were performed in the GloMax® Multi + Detection System (Promega, USA). Firefly luminescence reading for each sample was first normalised to *Renilla* luminescence reading as the internal control of transfection efficiency, before it was further normalised to the control cells transfected with pGL3-Basic plasmid for the comparison of promoter activities.

### Site-directed mutagenesis

Multiple base substitution mutations at the SRE site, proximal and distal CCAAT box sites were created in the *fads2*-244 plasmid employing the Muta-Direct^TM^ Site-Directed Mutagenesis Kit (iNtRON, Korea) following the manufacturer’s instructions. Complementary mutagenic primers (Supplementary data 5) used in the mutagenesis were designed using web-based programme PrimerX (http://www.bioinformatics.org/primerx/). Multiple site mutations were carried out one after another by using previously constructed mutant plasmids as templates.

### Electrophoretic mobility shift assay

Prior to electrophoretic mobility shift assay (EMSA), 3′-end of each complementary strand of the oligonucleotides (Supplementary data 6) were labelled with biotin using the Biotin 3′ End DNA Labelling Kit (Thermo Scientific, USA) following descriptions of the manufacturer. Subsequently, biotin-labelled double-stranded oligonucleotides (10 μM) were generated by mixing equal amounts of each complementary strand of the resulted oligonucleotides at least 1 h prior to EMSA. For non-labelled competition double-stranded oligonucleotides, equimolar of each complementary strand of oligonucleotides (10 µM) were added into 1X annealing buffer containing 10 mM Tris (pH 7.5), 1 mM EDTA and 50 mM NaCl, and the mixture was heated in the Thermomix (Eppendorf, Germany) for 5 min at 95 °C before gradually cooling down to room temperature over a period of approximately 1 h. Nuclear extract was prepared from the transfected ZFL cells using the NE-PER® Nuclear and Cytoplasmic Extraction Reagents (Thermo Scientific, USA) according to the manufacturer’s instructions. For EMSA, the LightShift® Chemiluminescent EMSA Kit (Thermo Scientific, USA) was used and each binding reaction containing 1X binding buffer, 1 µg Poly (dI·dC), 5% (v/v) glycerol, 0.05% (v/v) NP-40, 50 mM KCl, 5 mM MgCl2, 20 mM EDTA, 5 µl nuclear extract and 100 fmol biotin-labelled oligonucleotides in a total volume of 20 µl ddH2O was prepared. For the negative control binding reaction, nuclear extract was excluded, and 100-fold molar excess of non-labelled oligonucleotides was included to the competition binding reaction. The DNA-protein binding complexes were then incubated at room temperature for 30 min before size-fractioning on a 5% (v/v) non-denaturing polyacrylamide gel at 4 °C in the Mini-PROTEAN II Slab Electrophoresis Cell (Bio-Rad, USA). Finally, biotin-labelled DNA was detected with the aid of Chemiluminescent Nucleic Acid Detection Module (Thermo Scientific, USA) and luminescent signal was captured using the CCD camera (Bio-Rad, USA).

### GFP promoter-reporter plasmid construction

DNA fragments from the *fads2*−244 and −1735 promoter-luciferase reporter plasmids were PCR amplified using both forward and reverse primers containing the restriction site for *Xho*I and *BamH*I (Supplementary data 3), respectively. The resulted PCR products were gel-purified and cloned into the pZsGreen1-1 GFP promoter-reporter plasmid.

### Transient expression of GFP promoter-reporter plasmid in zebrafish embryo

*In vivo* promoter activity of both promoter fragments was determined by microinjection of GFP promoter-reporter plasmids into zebrafish embryos, respectively. Prior to microinjection, GFP promoter-reporter plasmids were linearised with the restriction enzymes *XhoI* and *NotI*. Each pZsGreen1-1 GFP promoter-reporter construct was adjusted to 10 ng/µl with dH_2_O containing 1X Danieau’s buffer and 0.2% phenol red. Approximately, 4.6 nl of the DNA solution was delivered into embryos at early one-cell stage by using the Nanoliter 2000 Microinjector (World Precision Instrument, USA). The injected embryos were incubated in E3 medium at 28.5 °C. The embryos were periodically monitored under the MVX10 Fluorescence Macro Zoom Microscope equipped with ColorView III Soft Imaging System (Olympus, Japan) until 120 hpf. For cryosectioning, zebrafish young larvae were fixed with 4% paraformaldehyde, treated and sectioned at a thickness of 20 µm using the Leica CM1850 UV Cryostat (Leica Biosystems, Germany).

## Electronic supplementary material

Supplementary Datasets
