# Supplementary material for: Transcriptional activation of zebrafish fads2 promoter and its transient transgene expression in yolk syncytial layer of zebrafish embryos
Source: Sci Rep. 2018 Mar 1;8:3874. doi: 10.1038/s41598-018-22157-4 (PMC5832746; doi:10.1038/s41598-018-22157-4)
Supplement: Supplementary file 1 — Supplementary Datasets [file 41598_2018_22157_MOESM1_ESM.doc]

**Transcriptional activation of zebrafish *fads2* promoter and its transient transgene expression in yolk syncytial layer of zebrafish embryos.**

**Shu Shen Tay^1^, Meng-Kiat Kuah^2^, Alexander Chong Shu-Chien^1,2*^**

**^1^ School of Biological Sciences, Universiti Sains Malaysia, 11800, Minden, Penang, Malaysia.**

**^2^ Centre for Chemical Biology, Universiti Sains Malaysia, Sains@USM, Block B No. 10, Persiaran Bukit Jambul, 11900, Bayan Lepas, Penang, Malaysia.**

***Corresponding author**

**email:** [**alex@usm.my**](mailto:alex@usm.my)

**Supplementary data 1**

**DNA sequence of the zebrafish (*Danio rerio*) *fads2* gene promoter region.** It contains 1735 nucleotides upstream of the transcription start site (TSS) and 19 nucleotides from the 5’-UTR region. The transcription initiation element, a classic TATA box is underlined. The transcription start site, a cytosine designating as +1 is indicated by a bent arrow. Forward primers used in the generation of 5’–deletion fragments are shaded in grey and the arrowheads above denote their positions relative to the TSS.

► -1735

-1735 ACGATGTTTA CTGCAATTTA GGTCACATTA TGCACTTTTT ATTATTGTAA ATGTAATGAT

-1675 ATATTTCTTA TAACCAATTT ATAGGTTTTA TATATGTAAT TATGCAGTAT TTTAAAATGT

-1615 TTTCCACACA AAAACAAAAT CCTATCAACT TTTTTTTACA TTTAACAGAA AATACATTGT

-1555 ATTTTTGTAT TATAATAAAT GTAAGAGTGT TTTTACAAGC TATGCATGAC ATTTATTGTG

-1495 ATTTAGGTAA CCGAATGTAC TTTTTTTATG TAAATGCAAC AATATATTAC TTATAATCAA

-1435 TTTATAGGTT TTGTATGTGT AATTGTGCAT TATTTTAAAA TGTTTTCCAC ACAAAGACAA

-1375 AACCCAAGCA AATCTGTCCT TTTAATTTAA CAGAAAATAA TACTAATTAC TATTACTAAT

-1315 ATAATACTAT ATTTGCATAT ACTAATGAAT GTAAGAGTGC TTTTACAAGC GATGTATGAC

-1255 ATTTAATGCA ATTAAGGTAA CTGAATGAAC TTTTTTAATG TAAATGCAAC TATATATTTG

► -1145

-1195 TTATAACCAA TTTATAGGTT TTGTATGTGT AATTATGCAG TATTTTAAAA TGTTTTCCAC

-1135 ACAATCCTAT CAACTTTCTT ATTTACATTT AACAGAAAAT ATTCTGTATT TTTGTATTCT

-1075 ACTGAACGAA GAGTGTTTTA CAAGCTATGT ACAACGTTTA ATGCGATTTA CATAACCAAA

-1015 TGCACTTTTT ATTCATGTAA ATACAACGAT ATATTACTTT TAAGCAAGGT ATAGGTTTTG

-955 CATGTGTACT TATGAAGTAT TTTAAAAGGT TTTCCACACA AACACAAAAT CCTATCACTT

-895 TTTTTTACAT TTAACAGAAA ATACACTGTA TTTATGTATT CTAATGAATG TAAGCGTGTT

-835 TTTACAAGCT ATGTACGACG TAATGCGATT TATGTCAGTG AATGCACTTT CTATTCATGT

-775 AAATGCAACG ATATACTACT TAAGAGCAAT TTATAGATTT TGTATGTGTA ATTATGCATT

-715 ACTTTTTATG TTTTTCACAC AAAGACAAAA CCCTATCAAA GTATTTTTAA TAAAACAAAA

-655 AATTCACTAT ATTTACTTAT TCTAAGGGGT TTTTATCCTC TTATTTTTGG ATTTACAAGC

► -586

-595 TTAACTTTGG ACTTCTCAAT GCAAAGGAAA AACCACAGAC CCAAACCTGC TCGTTTTTTT

-535 GCACTATCCT TTACGATATA CTGTATTTTT AGCATGTTCA CATGTTTAAC AAGAACGTTT

-475 ACAGTTAATT TTCGAGCATT TATTCTCTGA TAAGCTCCTA ATAAAACAAG ATAAGATAAT

-415 TAGGCTGCTC ATAAATGCTC GTTTGATGCA GGTATTGGAT CATTTCCCTT CACAACTAAT

-355 TTTTACACAT GAATTAGGTC ACCAAGTGCA CTTTTTACAT GGTAAATGCA AAAATATATT

► -244

-295 ACATATAAAC ATTATTTATA TGTTTTGTTT GTATAATGTT CGTTATTTTA TCGCCGTCAT

-235 TGTTTTCTGT TACCTCACTG ATGTAGAACT CGAACCTGAG TAACTCTTCT CTTTATCTCG

► -161

-175 CGTTTTCCCC GCCCTTCCAA AGTTCTCTCT GTGCTCCCAT TGGCTGACAG TCCGGAGACT

► -67


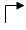
 -115 CTCTCAGAGA CGCGCGCCGA TTGGCTGCTG GAGCTCGAAT GATCTGTTCG GAATTCAGCA

**TATA box**

-55 GAGGTACAAG TGATGTGAGT GAGTGTATAA GACAGTATGA GTCTCTCATC CGCTTCTCTT

+6 TCTCATCACA CACA

**Supplementary data 2**

**Schematic illustration of potential regulatory binding sites within -161/-67 bp of the zebrafish *fads2* promoter region.** Wild type (above) and mutated (below) sequences of the SRE, proximal CCAAT box (CATp) and distal CCAAT box (CATd) are shown, whereby the core sequences are underlined and mutated bases are indicated with bold letters in italic.

**SRE**

-82/-72

CTCGAATGATC

CTC***TTT***TGATC

**CATp**

-99/-89

CCGATTGGCTG

CCGAT***AAA***CTG

**CATd**

-140/-130

CCCATTGGCTG

CCCAT***AAA***CTG

-161

-67

+1

**Supplementary data 3**

**Primers used for the construction of *fads2* promoter-reporter plasmids.** Restriction sites are underlined in italic and small letters denotes the additional bases for restriction enzyme digestion.

| **Plasmid** | **Primer** | **Primer sequence (5’–3’)** |
| --- | --- | --- |
| pGL3 luciferase promoter-reporter plasmid | | |
| *fads2*-1735 | F1735 | gcta*GGTACC*ACGATGTTTACTGCAATTTAGG |
| *fads2*-1145 | F1145 | gcta*GGTACC*TGTTTTCCACACAATCCTATCAAC |
| *fads2*-586 | F586 | gcta*GGTACC*GACTTCTCAATGCAAAGGAAAAA |
| *fads2*-244 | F244 | gcta*GGTACC*CGCCGTCATTGTTTTCTGTT |
| *fads2*-161 | F161 | gcta*GGTACC*TTCCAAAGTTCTCTCTGTGCTC |
| *fads2*-67 | F67 | gcta*GGTACC*CGGAATTCAGCAGAGGTACAAG |
|  | R19 | gcta*CTCGAG*TGTGTGTGATGAGAAAGAGAAGC |
|  |  |  |
| pZsGreen1-1 GFP promoter-reporter plasmid | | |
| *pZs*-1735 | F1735G | gcta*CTCGAG*ACGATGTTTACTGCAATTTAGG |
| *pZs*-244 | F244G | gcta*CTCGAG*CGCCGTCATTGTTTTCTGTT |
|  | R19G | gcta*GGATCC*TGTGTGTGATGAGAAAGAGAAGC |

**Supplementary data 4**

**Primers used for the construction of pcDNA3.1*-*nSrebp expression plasmids.** Restriction sites are underlined in italic and small letters denotes the additional bases for restriction enzyme digestion.

| **Plasmid** | **Primer** | **Primer sequence (5’–3’)** |
| --- | --- | --- |
| pcDNA3.1-nSrebp expression plasmid | | |
| nSrebp1 | nS1For | gcta*GGTACC*ATGAATCTGTCTTTTGACGACA |
|  | nS1Rev | gcta*CTCGAG*CTACAGAGCCATGCGGGCGGT |
| nSrebp2 | nS2For | gcta*GGTACC*ATGGACGCCTCGGAGTTTATGG |
|  | nS2Rev | gcta*CTCGAG*CTACAGCAGCAGACGAGAGCGGTCC |

**Supplementary data 5**

**Primers used for mutagenesis of the oligonucleotides.** Mismatched nucleotides are underlined in italic.

| **Binding site** | **Primer** | **Primer sequence (5’–3’)** |
| --- | --- | --- |
| Proximal CCAAT box | Forward | CTCGGAGACGCGCGCCGAT*AAA*CTGCTGGAGCTCGAATGATC |
|  | Reverse | GATCATTCGAGCTCCAGCAG*TTT*ATCGGCGCGCGTCTCCGAG |
|  |  |  |
| Distal CCAAT box | Forward | CTCTCTGTGCTCCCAT*AAA*CTGACAGTCCGCGAG |
|  | Reverse | CTCGCGGACTGTCAG*TTT*ATGGGAGCACAGAGAG |
|  |  |  |
| SRE | Forward | ATTGGCTGCTGGAGCTC*TTT*TGATCTGTTCGGAATTCAGCAG |
|  | Reverse | CTGCTGAATTCCGAACAGATCA*AAA*GAGCTCCAGCAGCCAAT |

**Supplementary data 6**

**Oligonucleotides used for EMSA.** Core binding sites are underlined and bold letters in italic indicates mismatched nucleotides.

| **Oligonucleotide** | **Primer sequence (5’–3’)** |
| --- | --- |
| *Proximal CCAAT box* | |
| CATp wt For | CTCAGAGACGCGCGCCGATTGGCTGCTGGAGCTCGAATGATC |
| CATp wt Rev | GATCATTCGAGCTCCAGCAGCCAATCGGCGCGCGTCTCTGAG |
| CATp mut For | CTCAGAGACGCGCGCCGAT***AAA***CTGCTGGAGCTCGAATGATC |
| CATp mut Rev | GATCATTCGAGCTCCAGAG***TTT***ATCGGCGCGCGTCTCTGAG |
|  |  |
| *Distal CCAAT box* | |
| CATd wt For | CTCTCTGTGCTCCCATTGGCTGACAGTCCGGAG |
| CATd wt Rev | CTCCGGACTGTCAGCCAATGGGAGCACAGAGAG |
| CATd mut For | CTCTCTGTGCTCCCAT***AAA***CTGACAGTCCGGAG |
| CATd mut Rev | CTCCGGACTGTCAG***TTT***ATGGGAGCACAGAGAG |
|  |  |
| *SRE and proximal CCAAT box* | |
| SRE-CAT wt For | CGCGCGCCGATTGGCTGCTGGAGCTCGAATGATCTGTTCGG |
| SRE-CAT wt Rev | CCGAACAGATCATTCGAGCTCCAGCAGCCAATCGGCGCGCG |
| SREmut-CATwt For | CGCGCGCCGATTGGCTGCTGGAGCTC***TTT***TGATCTGTTCGG |
| SREmut-CATwt Rev | CCGAACAGATCA***AAA***GAGCTCCAGCAGCCAATCGGCGCGCG |
| SREwt-CATmut For | CGCGCGCCGAT***AAA***CTGCTGGAGCTCGAATGATCTGTTCGG |
| SREwt-CATmut Rev | CCGAACAGATCATTCGAGCTCCAGCAG***TTT***ATCGGCGCGCG |
